# Individual variation in alpha neurofeedback training efficacy predicts pain modulation

**DOI:** 10.1016/j.nicl.2020.102454

**Published:** 2020-09-29

**Authors:** Weiwei Peng, Yilin Zhan, Yali Jiang, Wenya Nan, Roi Cohen Kadosh, Feng Wan

**Affiliations:** a School of Psychology, Shenzhen University, Shenzhen, Guangdong, China; b Shenzhen Key Laboratory of Affective and Social Cognitive Science, Shenzhen University, Shenzhen, Guangdong, China; c Department of Psychology, Shanghai Normal University, Shanghai, China; d Wellcome Centre for Integrative Neuroimaging, Department of Experimental Psychology, University of Oxford, Oxford, United Kingdom; e Department of Electrical and Computer Engineering, Faculty of Science and Technology, University of Macau, Macau, China; f Centre for Cognitive and Brain Sciences, Institute of Collaborative Innovation, University of Macau, Macau, China

**Keywords:** Pain, Neurofeedback, Sensorimotor α-oscillation, Pain threshold, Pain intensity, Unpleasantness

## Abstract

Studies have shown an association between sensorimotor α-oscillation and pain perception. It suggests the potential use of neurofeedback (NFB) training for pain modulation through modifying sensorimotor α-oscillation. Here, a single-session NFB training protocol targeted on increasing sensorimotor α-oscillations was applied to forty-five healthy participants. Pain thresholds to nociceptive laser stimulations and pain ratings (intensity and unpleasantness) to identical laser painful stimulations were assessed immediately before and after NFB training. Participants had larger pain thresholds, but rated the identical painful laser stimulation as more unpleasant after NFB training. These pain measurements were further compared between participants with high or low NFB training efficacy that was quantified as the regression slope of α-oscillation throughout the ten training blocks. A significant increase in pain thresholds was observed among participants with high-efficacy; whereas a significant increase in pain ratings was observed among participants with low-efficacy. These results suggested that NFB training decreased the sensory-discriminative aspect of pain, but increased the affective-motivational aspect of pain, whereas both pain modulations were dependent upon the NFB training efficacy. Importantly, correlation analysis across all participants revealed that a greater NFB training efficacy predicted a greater increase in pain thresholds particularly at hand contralateral to NFB target site, but no significant correlation was observed between NFB training efficacy and modulation on pain ratings. It thus provided causal evidence for a link between sensorimotor α-oscillation and the sensory-discriminative aspect of pain, and highlighted the need for personalized neurofeedback for the benefits on pain modulation at the individual level. Future studies can adopt a double-blind sham-controlled protocol to validate NFB training induced pain modulation.

## Highlights

• Sensorimotor alpha neurofeedback training effect on pain perception was assessed.
• Neurofeedback training decreased the sensory-discriminative aspect of pain.
• Neurofeedback training increased the affective-motivational aspect of pain.
• Pain modulation by neurofeedback training was dependent upon the training efficacy.
• Neurofeedback training efficacy predicted sensory-discriminative pain modulation.

## 1 Introduction

Oscillations of brain activities within the alpha-frequency band (8–12 Hz, α-oscillations) is generally thought to reflect mechanisms that inhibit processing of irrelevant sensory and task information by gating the information flow across different brain regions (Jensen and Mazaheri, 2010, Klimesch, 2012). The amplitude of α-oscillations has been interpreted as a measure of local neuronal ensemble excitability (Lange et al., 2013, Sauseng et al., 2009). For example, suppression of sensorimotor α-oscillation induced by nociceptive stimulation is thought to reflect the excitability of the somatosensory cortex (Hu et al., 2013, Ploner et al., 2006). Previous studies have documented the association between spontaneous α-oscillations and the perception of both experimental and clinical pain (Peng et al., 2015, Ploner et al., 2017). For instance, the amplitude of spontaneous α-oscillations over sensorimotor cortex was negatively correlated with pain perception (Babiloni et al., 2006, Tu et al., 2016). Abnormal spontaneous α-oscillations have been observed among patients with chronic pain, a phenomenon that has been interpreted as dysfunctional cortical inhibition (Ahn et al., 2019, Kim et al., 2013, Pinheiro et al., 2016, Ye et al., 2019). The relationship between α-oscillation amplitude and pain perception suggests that a neuromodulation technique that can alter sensorimotor α-oscillation has the potential to modulate pain perception.

Neurofeedback (NFB) is a type of biofeedback in which participants are provided information about their brain activities through visual or auditory representations. It has been adopted as a means for self-regulating the putative neural substrates that underlie specific behaviors or psychophysiology (Sitaram et al., 2017). NFB has been used for the treatment of clinical pain by facilitating α-oscillations (i.e., increasing their amplitude) (Al-Taleb et al., 2019, Hasan et al., 2016, Hassan et al., 2015, Jensen et al., 2013a, Jensen et al., 2013b, Vuckovic et al., 2019). For instance, patients with central neuropathic pain reported decreased pain intensity after completing a long-term NFB training therapy that targeted facilitation of sensorimotor α-oscillations and reduction of theta (4–8 Hz, θ-oscillations) and beta (20–30 Hz, β-oscillations) band brain activities. Indeed, this pain-modulatory effect lasted at least one month after therapy had been completed (Hassan et al., 2015). Nevertheless, these studies adopted NFB protocols that simultaneously regulated several different bands of brain activity, rather than only affecting α-oscillations (Al-Taleb et al., 2019, Hassan et al., 2015, Jensen et al., 2013a, Vuckovic et al., 2019). Given the close association between sensorimotor α-oscillation and subjective pain perception (Babiloni et al., 2006, Tu et al., 2016), it is likely that NFB training targeted on increasing sensorimotor α-oscillation alone would preferably attenuate pain perception.

The present study therefore aimed to directly assess whether or not NFB training that facilitates only sensorimotor α-oscillations can modulate pain perception. If so, we also wanted to determine whether the pain modulatory effect can be predicted by the training efficacy. We adopted a single-session NFB protocol focused on increasing α-oscillation amplitude at sensorimotor electrodes. The efficacy of self-regulating the α-oscillations was quantified as the slope of the linear regression of α-oscillation data, specifically at training target site, throughout the 10 training blocks within the single NFB training session. Immediately before and after the NFB training session, individual pain perception to experimental nociceptive laser stimuli was quantified by assessing pain thresholds and obtaining subjective pain ratings to identical painful stimulations. Given the relationship between brain α-oscillations and pain perception, we hypothesized that after NFB training participants would have larger pain thresholds and rated the pain stimuli as less painful. Further, we hypothesized that the NFB training efficacy would predict the degree to which these pain measurements changed.

## 2 Materials and methods

### 2.1 Participants

Forty-five adults (29 males and 16 females; mean age: 21.80 ± 0.30 [SEM] years; age range: 18–27 years) from Shenzhen University (Guangdong, China) participated in this study as paid volunteers. None of the participants had been previously diagnosed with a medical, neurological, or psychiatric disorder. All participants were right-handed and had normal or corrected-to-normal vision and hearing. While this study aimed to assess the relationship between NFB training efficacy and pain modulation, previous studies have reported an average effect size of *r* = 0.40 for the correlation between NFB training induced EEG and behavioral changes (Nan et al., 2012, Nan et al., 2020, Schabus et al., 2014). According to G*Power software (Faul et al., 2007), a sample size between 34 (for one-tailed correlation analyses) and 44 (for two-tailed correlation analyses) is appropriate to detect an effect size of *r* = 0.40, at significance level of 0.05 with 80% power. Therefore, our sample size (*n* = 45) was appropriate for the statistical analyses we planned to perform. The experiment was carried out in accordance with Declaration of Helsinki. All participants gave their written informed consent before the experiments and all experimental procedures were approved by the local ethics committee. The consensus on the reporting and experimental design of clinical and cognitive–behavioral NFB studies (CRED–nf checklist, Ros et al., 2020) was included in the supplementary section.

### 2.2 NFB training

**Fig. 1:**
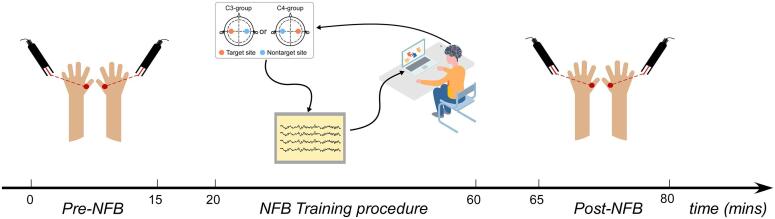
Schematic illustration of the experimental procedure.

A commercial NFB system (NeXus-10 MKII, MindMedia BV, Netherlands) and software (Biotrace+, MindMedia BV, Netherlands) were used for NFB training. As shown in Fig. 1, the target site for training was at either the C3 (C3-group) or C4 (C4-group) electrode on the international 10–20 system. The contralateral electrode (C4 for the C3-group; C3 for the C4-group) was the nontarget control electrode for offline data analysis. Participants were blinded to the NFB training target site (C3 or C4), but experimenters were not blinded due to the NFB software limitation. Throughout the training procedure, electroencephalographic (EEG) signals at both target and nontarget sites were acquired at a sampling rate of 256 Hz. The ground was located at the forehead and the reference for each recording location was the contralateral mastoid.

Each participant completed a single-session of NFB training. The session comprised 10 training blocks, with each block lasting 3 min and an inter-block-interval of about 2 min. Therefore, training overall lasted about 48 min. Before training began, we recorded 2-minute of eyes-open resting EEG. The EEG feature for the online NFB training was the α-oscillation amplitude at the target site (C3 for the C3-group; C4 for the C4-group). For real-time estimation of the feature, the EEG signal was bandpass filtered within 8–12 Hz (infinite impulse response Butterworth bandpass 3rd order), and the root mean square (RMS) of the resulting signal within rolling 0.25-second windows was calculated.

A visual game (puzzles) was used to present feedback information about the EEG signals at the target electrode. Participants were instructed to move the puzzle pieces toward the target area according to the EEG feedback signals, but they were not provided with any explicit instructions regarding strategy. During the training block, when the online α-oscillation amplitude was above the pre-set threshold, the puzzle piece would move toward the target location, provided that the high frequency (75–100 Hz) EEG amplitude was below 5 μV. That is, participants were not rewarded if high frequency EEG amplitude was above 5 μV, even when the α-oscillation amplitude was above the pre-set threshold. Here, the control of high frequency EEG amplitude was to minimize the influence of muscle activity on the feedback EEG signals of NFB (Kober et al., 2015a). The threshold of the first training block was set to the α-oscillation amplitude during the eyes-open resting EEG recording performed before the training. In the remaining blocks, the threshold for each block was updated according to the percentage of time α-oscillations were above threshold in the previous training block: if the percentage was above 60% (or below 40%), the reward threshold would be adjusted by increasing (or decreasing) the previous threshold by 0.5 μV. If the percentage was in the range of 40%−60%, the reward threshold would remain unadjusted.

### 2.3 Laser stimulation

Nociceptive-specific radiant-heat stimuli were generated by an infrared neodymium yttrium aluminum perovskite laser with a wavelength of 1.34 μm and a pulse duration of 4 ms (Electronic Engineering, Italy). At this wavelength and pulse duration, laser stimuli directly activate nociceptive terminals in the most superficial layers of the skin in a synchronized fashion (Iannetti et al., 2004). A He–Ne laser pointed toward the area to be stimulated. The laser beam was transmitted via an optic fiber and its diameter was set at approximately 7 mm by focusing lenses. Laser pulses were delivered to a square area (4 cm × 4 cm) on the hand dorsum. After each stimulation, the beam target was shifted by at least 1 cm in a random direction within the square area to avoid nociceptor fatigue or sensitization.

### 2.4 Experimental procedure

As illustrated in Fig. 1, participants were randomly assigned to either the C3-group (*n* = 21; NFB target site = C3 electrode) or the C4-group (*n* = 24; NFB target site = C4 electrode). Impedances at both target and nontarget sites were kept below 5 kΩ. The experiment consisted of three phases, separated by a 3-minute break: Pre-NFB (~15  min), NFB training (~48  min), and Post-NFB (~15  min).

In both Pre- and Post-NFB sessions, participants were instructed to complete the pain assessment for both hands. This included a pain-threshold determination and pain-intensity ratings in response to suprathreshold nociceptive laser stimulation. For assessing pain threshold, the energy of laser stimulation started at 0.5 J and increased in 0.25-J steps, with a maximum possible stimulation energy of 3.5 J. After each stimulation, the participant was instructed to report whether they detected any pain or not. The interval between consecutive trials varied randomly between 17 and 22 s. The energy with which the laser stimulation could elicit a reliable pain sensation (pain is felt in 5 out of 10 trials at a single intensity) was recorded as the pain threshold. This procedure was repeated twice for each hand, with about 2 min between the repetitions.

To assess how painful a given pain stimulus was, participants rated the intensity of supra-threshold pain stimulation. Nociceptive laser stimuli at an energy of 3 J were delivered to the dorsum of both hands (10 stimuli per hand). The inter-stimulus-interval varied randomly between 17 and 22  s. The hand order was pseudorandomized, with the constraint that no more than two stimuli were delivered consecutively to the same hand. Approximately 3  s after each laser stimulus, participants were asked to verbally report both perceived pain intensity and unpleasantness on a numerical rating scale (NRS) ranging from 0 (no pain/unpleasantness) to 10 (pain/unpleasantness as bad as it could be).

Participants completed a 48-minute-long NFB training procedure, with training target site located at either C3 (C3-group) or C4 (C4-group). EEG signals at the electrode contralateral to the target site were simultaneously recorded as the nontarget site (C4 for C3-group, C3 for C4-group). A visual game was used to present EEG feedback from the target site. Participants were not provided with any explicit instructions or strategy on how to adjust their brain activity. About 3 min before and after the NFB training procedure, pain measurements was assessed by delivering laser stimulation at the dorsum of both hands. Specifically, pain thresholds and subjective pain ratings (intensity and unpleasantness) to supra-threshold stimulation (at 3 J) were assessed.

### 2.5 EEG data analysis

The EEG data obtained during training was processed offline using custom scripts and the EEGLAB toolbox (Delorme and Makeig, 2004) running under MATLAB (The Mathworks, Natick, MA, USA). For each NFB training block and electrode location (target and nontarget sites), the continuous EEG data were bandpass filtered from 2 to 20 Hz using a Hamming windowed Finite Impulse Response (FIR) sinc filter through the eegfiltnew function, and further were segmented into 2-second epochs with an overlap of 0.5 s. For each epoch and frequency point, the log-transformed power spectrum density (PSD) was computed using the spectopo function. Epochs with log-transformed PSD at any frequency point within 2–4 Hz greater than 2.5 standard deviations above the mean of all epochs were identified as outliers and rejected (Escolano et al., 2014), because ocular artifacts are dominant at frequencies below 4 Hz (Fatourechi et al., 2007). Then, for each participant, NFB training block, and electrode location, α-oscillation amplitude was calculated as the mean spectrum amplitude within 8–12 Hz across the artifact-free epochs.

During the NFB training procedure, while all participants received real NFB training of α-oscillation at the target site, EEG signals at target and nontarget sites were simultaneously recorded, and the target site was blinded to the participants. Here, the nontarget site could be considered as a within-participant control site. The lateralization index (LI) of α-oscillation amplitude between target and nontarget sites was a normalized α-oscillation at the target site relative to the nontarget site. For each participant and NFB training block, LI was calculated according to the following equation: LI = (Target – Nontarget) / (Target + Nontarget), where Target represents the α-oscillation amplitude at the target site, and Nontarget represents the α-oscillation amplitude at the nontarget site. A positive or a negative LI value indicates an increase or a decrease of α-oscillation amplitude at the target site relative to that at the nontarget site.

NFB training efficacy was often quantified using the slope of the linear regression of EEG oscillation throughout the training blocks (Berger and Davelaar, 2018, Kober et al., 2018, Mottaz et al., 2015, Nan et al., 2015). In addition, previous studies (see Gruzelier, 2014 for a review) have shown that NFB training would induce changes in brain activities not only at the target site but also at the nontarget site. Here, we characterized NFB training efficacy using the slope of linear regression of α-oscillation LI throughout the NFB training blocks, as it can reflect the degree of which NFB training influenced the α-oscillation amplitudes with topographical specificity at the target site. When computing the regression slope of α-oscillation LI for each participant, the independent variable was the block number ranging from 1 to 10, and the dependent variable was the α-oscillation LI for the corresponding NFB training block. Indeed, this approach could help strength the support that the observed changes of pain measurements after NFB training, was associated with a real NFB training effect on the brain activity of the NFB target site.

### 2.6 Statistical analysis

For statistical analysis, we used the statistics toolbox running under MATLAB (The Mathworks, Natick, MA, USA) as well as SPSS 24 (IBM Corp, Armonk, NY, USA).

First, we investigated the overall NFB training effects on EEG and pain measures based on all participants (*n* = 45). The amplitude of α-oscillation throughout the NFB training blocks was compared using a repeated-measures analysis of variance (ANOVA) with Block (1–10) and Location (target or nontarget site) as the within-participant factors. Pain measurements (pain thresholds, as well as pain intensity and unpleasantness ratings) were compared using a repeated-measures ANOVA with two within-participant factors of Stimulation Site (contralateral or ipsilateral to the target site) and Session (pre- or post-NFB session). Post hoc comparisons were performed if any significant main effect or interaction effect was observed.

Second, we assessed whether the change of pain measurement after NFB training was dependent upon the efficacy during the NFB training, which was indexed by the regression slope of α-oscillation LI throughout the ten NFB training blocks. Participants were assigned to high- or low-efficacy groups if their LI slopes respectively landed in the upper and lower quartiles of the overall distribution from all participants (*n* = 45). Then, we applied the mixed-design three-way ANOVA with a between-participant factor of Group (high- vs. low-efficacy group), and two within-participant factors of Stimulation Site (contralateral vs. ipsilateral to target site) and Session (pre- vs. post-NFB session) on pain measurements. We performed post hoc comparisons if any significant main effect or interaction effect was observed.

If any pain measurement change was dependent upon NFB training efficacy, we performed a correlation analysis to assess whether the change of pain measurement (difference between pre- and post-NFB sessions) was predicted by the NFB training efficacy. Bivariate outliers were detected by median absolute deviation-median rule using a free MATLAB-based toolbox (Pernet et al., 2013) and removed before the correlation analysis. One-tailed Spearman correlation was adopted as we expected that individuals with greater NFB training efficacy tend to exhibit greater decrease in pain perception. Benjamini-Hochberg false discovery rate (FDR) procedure was applied to account for the multiple comparison problem (Benjamini and Hochberg, 1995).

## 3 Results

### 3.1 EEG α-oscillation

**Fig. 2:**
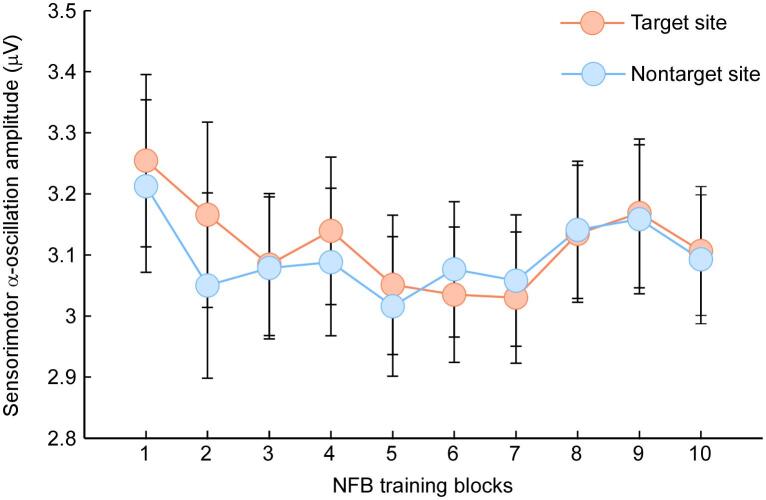
Grand average sensorimotor α-oscillation amplitudes at target and nontarget sites throughout the NFB training blocks (1–10). Data points are the means and error bars are the SEM.

Grand averages of EEG α-oscillation amplitudes at the target and nontarget sites are shown in Fig. 2. The repeated-measures ANOVA showed no significant main effect of Block (*F_9,396_* = 1.40, *p* = 0.19, η_p_^2^ = 0.031) or Location (*F_1,44_* = 0.08, *p* = 0.78, η_p_^2^ = 0.002), and no significant interaction between the two (*F_9,396_* = 0.87, *p* = 0.55, η_p_^2^ = 0.019). Thus, no significant NFB training effect was observed, suggesting that the used NFB training protocol did not have an overwhelming influence on α-oscillation amplitude at the group-level. This could have been arisen from the great variability existed between the participants. For an illustration purpose, the EEG α-oscillation from two participants who respectively exhibited high and low NFB training efficacy were displayed in the Fig. S1 (see Supplementary Materials).

### 3.2 Pain measurements

**Fig. 3:**
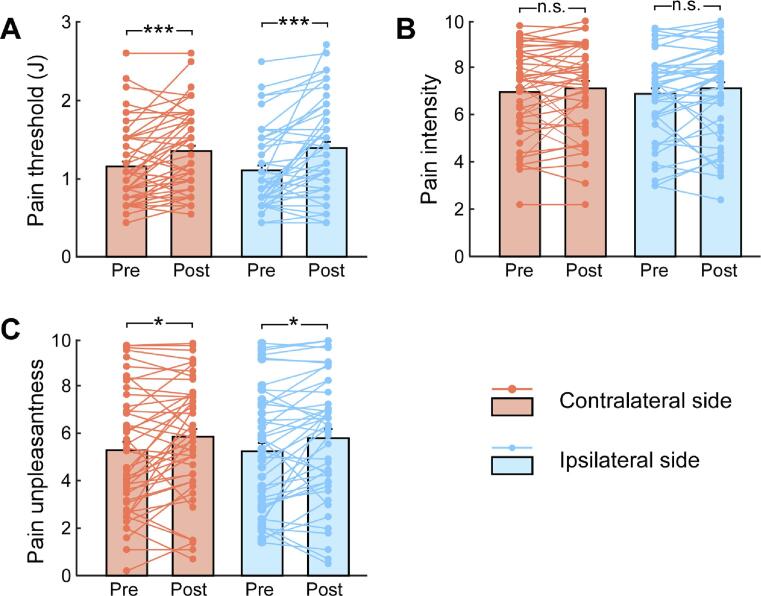
Pain measurements before and after NFB training. Graphs show pain measurements in both pre- and post-NFB sessions, including the pain threshold (A), pain intensity (B) and unpleasantness (C) ratings to suprahthreshold painful stimuli at hands contralateral or ipsilateral to NFB target site. Bars represent Mean ± SEM; symbols represent individual repeated-measures data (45 participants). n.s.: *p* > 0.05; *: *p* < 0.05; ***: *p* < 0.001; paired-sample *t*-test between pre- and post-NFB sessions.

Grand average pain measurements are summarized in the Fig. 3, and their statistical comparisons are summarized in Table S1 (see Supplementary Materials). Specifically, pain thresholds were only significantly modulated by Session (*F_1,44_* = 25.51, *p* < 0.001, η_p_^2^ = 0.37), indicating that pain thresholds were significantly larger in the post-NFB session than in the pre-NFB session. As shown in the Fig. 3A, pain thresholds significantly increased in the post-NFB session for hands contralateral (*t_44_* = 3.93, *p* < 0.001) and ipsilateral (*t_44_* = 4.54, *p* < 0.001) to the NFB target site. We did not observe significant main effects or interactions for pain intensity ratings (*p* > 0.05 for all comparisons, Fig. 3B). Pain unpleasantness ratings were also only modulated significantly by Session (*F_1,44_* = 7.24, *p* = 0.010, η_p_^2^ = 0.14), such that unpleasantness ratings in the post-NFB session were greater than those in the pre-NFB session. As shown in Fig. 3C, unpleasantness ratings significantly increased in the post-NFB session for hands contralateral (*t_44_* = 2.55, *p* = 0.014) and ipsilateral (*t_44_* = 2.68, *p* = 0.01) to the NFB target site. These results on pain measurements suggest that in the post-NFB session, participants had larger pain thresholds, but felt that suprathreshold nociceptive laser stimuli were more unpleasant than they had been in the pre-NFB session.

### 3.3 Relationship between NFB training efficacy and changes of pain measurements

**Fig. 4:**
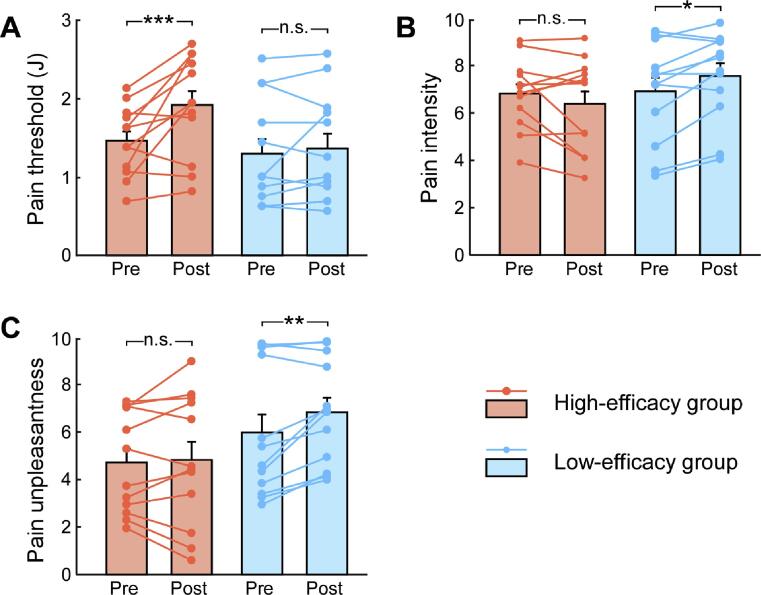
Pain measurements before and after NFB training for participants with high and low NFB training efficacy. Graphs show pain measurements in both pre- and post-NFB sessions, including the pain threshold (A), pain intensity (B) and unpleasantness (C) ratings to suprahthreshold painful stimuli for two groups of participants with high and low NFB training efficacy. Bars represent Mean ± SEM; symbols represent individual repeated-measures data (12 participants for each group). n.s.: *p* > 0.05; *: *p* < 0.05; **: *p* < 0.01; ***: *p* < 0.001; paired-sample *t*-test between pre- and post-NFB sessions.

According to the NFB training efficacy (quantified as the linear regression slope of α-oscillation LI throughout the NFB training blocks), there were 12 participants in the high-efficacy group (3 females, 21.50 ± 0.58 years) and 12 participants in the low-efficacy group (5 females, 21.25 ± 0.51 years). Groups did not differ in age (*t_22_* = 0.32, *p* = 0.75, independent-sample *t*-test) or sex (*χ*^2^ = 0.75, *p* = 0.39, Chi-Square test). The between-group statistical comparisons on pain measurement are summarized in Table S2 (see Supplementary Materials). Analysis on the pain threshold only revealed a significant interaction between Group and Session (*F_1,22_* = 5.45, η_p_^2^ = 0.199, *p* = 0.029). As shown in the Fig. 4A, participants in the high-efficacy group, pain thresholds were larger in the post-NFB session than that in the pre-NFB session (1.91 ± 0.19 vs. 1.46 ± 0.17, *p* = 0.001), whereas no significant difference was observed in the low-efficacy group (1.36 ± 0.19 vs. 1.29 ± 0.17, *p* = 0.57). These results indicated that the participants with high NFB training efficacy exhibited an increase of pain thresholds after NFB application, but not for participants with low NFB training efficacy.

Analysis on the ratings of pain intensity also revealed a significant interaction between Group and Session (*F_1,22_* = 8.01, η_p_^2^ = 0.27, *p* = 0.01). As shown in the Fig. 4B, for participants in the low-efficacy group, ratings of pain intensity was higher in the post-NFB session than those in the pre-NFB session (7.56 ± 0.55 vs. 6.91 ± 0.53, *p* = 0.025), whereas no significant difference was observed in the high-efficacy group (6.39 ± 0.55 vs. 6.82 ± 0.53, *p* = 0.13). For the ratings of pain unpleasantness, there was a marginal interaction between Group and Session (*F_1,22_* = 3.42, η_p_^2^ = 0.14, *p* = 0.078). As shown in the Fig. 4C, planned analysis showed that for participants in the low-efficacy group, ratings of pain unpleasantness were higher in the post-NFB session than those in the pre-NFB session (6.85 ± 0.73 vs. 5.99 ± 0.72, *p* = 0.007), whereas no significant difference was observed in the high-efficacy group (4.84 ± 0.73 vs. 4.73 ± 0.72, *p* = 0.72). These results indicated that participants with low NFB training efficacy reported more pain ratings (intensity and unpleasantness) to suprathreshold painful stimuli after NFB training, but not for those with high NFB training efficacy.

**Fig. 5:**
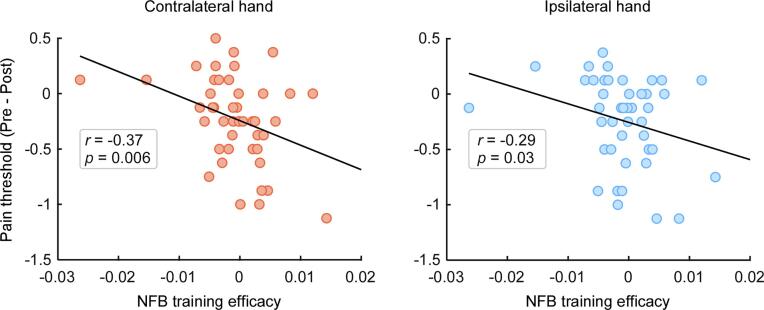
Across-participant correlations between NFB training efficacy and the change in pain threshold. Individual NFB training efficacy was quantified using the regression slopes for the α-oscillation LI, and the change in pain thresholds was quantified as the difference between pre-NFB and post-NFB sessions (pre-NFB minus post-NFB). Across all participants (*n* = 45), NFB training efficacy was correlated with the degree of change in pain thresholds at hands contralateral or ipsilateral to NFB target site. Each dot represents a single participant. The black line represents the best linear fit for the data.

Given that the change of pain thresholds and pain ratings was dependent on NFB training efficacy, we further assessed the correlations between NFB training efficacy and the change in pain measurement using Spearman correlation analysis. As shown in the Fig. 5, NFB training efficacy correlated significantly with the change in pain thresholds for the hand contralateral to the NFB target site (*r* = −0.37, *p* = 0.006) which passed the FDR correction. In contrast, the correlation between NFB training efficacy and pain threshold change for the hand ipsilateral to the NFB target site (*r* = −0.29, *p* = 0.03) did not survive after FDR correction. This suggests that a more effective NFB training (a steeper α-oscillation LI slope) reliably predicts a greater increase in pain thresholds during the post-NFB session, particularly at hand contralateral to the NFB target site. Nevertheless, we did not observe significant correlations between NFB training efficacy and the change in pain ratings to identical suprathreshold painful stimuli (*p* > 0.05 for all comparisons). It indicated that a greater functional inhibition topographical specifically at the NFB target site, predicts a larger increase in pain thresholds, particularly at the hand contralateral to NFB target site.

## 4 Discussion

The present study used a single-session NFB training protocol to facilitate sensorimotor α-oscillations in forty-five healthy participants. Individual NFB training efficacy was quantified using the regression slope of the α-oscillation LI throughout the 10 NFB training blocks, and degree of pain modulation was quantified by the change in pain thresholds and perceived pain ratings in response to identical painful stimuli. Overall, participants had larger pain thresholds, but reported more unpleasantness to identical painful stimuli in the post-NFB session as compared with the pre-NFB session. It indicates that NFB training decreased the sensory-discriminative aspect of pain, but increased the affective-motivational aspect of pain. We further performed subgroup analysis to compare the degree of pain modulation between two groups of participants with high or low NFB training efficacy. The significant increase in pain thresholds after NFB training was selectively applicable to the high-efficacy group; whereas the significant increase in pain ratings after NFB training was selectively applicable to the low-efficacy group. It suggests that the observed modulation on the sensory-discriminative and affective-motivational aspects of pain was dependent upon NFB training efficacy. Correlation analysis across all participants further confirmed that a greater NFB training efficacy predicts a greater increase in pain thresholds particularly at hand contralateral to NFB training site, thus providing evidence for a causal link between sensorimotor α-oscillations and the sensory-discriminative aspect of pain.

### 4.1 EEG α-oscillation and pain measurement

Pain related studies have documented that α-oscillation amplitude at sensorimotor regions modulates behavioral and neural responses to experimental painful stimuli such that greater amplitudes of spontaneous α-oscillations predict less pain perception (Babiloni et al., 2006, Peng et al., 2015, Ploner et al., 2017, Tu et al., 2016). This evidence leads to the hypothesis that neuromodualtory interventions that enhance sensorimotor α-oscillation can attenuate pain perception. Therefore, we used an NFB training protocol that targeted on increasing α-oscillation amplitude at sensorimotor electrodes (either C3 or C4) for the purpose of attenuating pain perception to nociceptive laser stimuli delivered to the dorsum of both hands. During the training session, participants were instructed to move the puzzles toward the target location according to real-time sensorimotor α-oscillation amplitude. At the group level, α-oscillation amplitudes did not increase either at target or nontarget site throughout the training blocks. This could have been attributed by the large inter-individual variability in NFB training efficacy (Alkoby et al., 2018, Baumeister et al., 2018, Reiner et al., 2018, Zuberer et al., 2015). Some participants can successfully increase the α-oscillation amplitude at the target site during the NFB training; whereas some participants cannot increase, or even decrease the α-oscillation amplitude at the target site (see Fig. S1 in the Supplementary Materials). Indeed, although many participants could successfully learn to self-regulate their brain activity through NFB training, 20% to 30%, or even up to 50% of participants could not, especially in single-session NFB training protocol (see Alkoby et al., 2018 for a review). The unsatisfactory NFB training for some participants could have arisen from the unsuccessful learning for the association between feedback EEG signals and visual representations. To overcome this problem, future studies can consider using a more simplified and intuitive representation of feedback EEG features, e.g. the size of a sphere or the height of a moving bar (Kober et al., 2018, Nan et al., 2012).

Immediately before and after the NFB training, pain thresholds to nociceptive laser stimuli, as well as subjective pain ratings to laser painful stimuli were assessed. Though pain undeniably has a sensory-discriminative aspect (such as quality and location), what makes it special is its affective-motivational quality of hurting which aspect makes us want to take protective action (Auvray et al., 2010, Fields, 1999). In particular, the pain threshold evaluates the sensory-discriminative aspect of pain, whereas pain unpleasantness rating evaluates the affective-motivational aspect of pain. Pain intensity ratings likely encompass multiple aspects of pain, as processing of pain intensity was reported to be associated with activation of a functionally diverse group of brain regions, including those important in sensation, affect, motor control and attention (Coghill et al., 1999). The comparison of pain measurements between pre- and post-NFB sessions revealed that participants overall had larger pain thresholds, but reported that the painful stimuli were significantly more unpleasant after NFB application. It indicates that NFB trainings decreased the sensory-discriminative aspect of pain (as indexed by the increased pain thresholds), but increased the affective-motivational aspect of pain (as indexed by the increased pain unpleasantness ratings).

### 4.2 Relationship between NFB training efficacy and pain modulation

We further assessed whether the modulation of pain perception was dependent upon the NFB training efficacy. Here, NFB training efficacy was quantified individually using the regression slope of α-oscillation LI throughout the 10 training blocks, as it characterizes how much NFB training influenced the α-oscillation amplitudes with topographical specificity at the target site relative to the nontarget site (as a within-participant control). Increased cortical α-oscillation amplitude has been related to cortical excitability such that increases in α-oscillation synchronization has been associated with a decrease in neuronal excitability (Lange et al., 2013) and the blood-oxygen-level-dependent signal (Scheeringa et al., 2011). Here, the α-oscillation LI regression slope can depict how NFB training influences sensorimotor cortical excitability specifically at the target site. A more positive LI slope indicated a greater functional inhibition at the target site compared with the nontarget site. According to the regression slope of α-oscillation LI, two groups of participants with high or low NFB training efficacy were identified (*n* = 12 for each group). Between-group comparisons showed that the modulation of pain perception on both sensory-discriminative and affective-motivational aspects was dependent upon NFB training efficacy: the increased pain thresholds after NFB training was selectively observed in the high-efficacy group, whereas the increased pain ratings after NFB training was selectively observed in the low-efficacy group. Therefore, if participants can effectively inhibit target site cortical excitability during the training session, they would have decreased pain perception at the sensory-discriminative aspect (as indexed by increased pain thresholds). Otherwise, affective-motivational responses to painful events would be exacerbated, which could be resulting from psychosocial factors such as negative mood and emotion during the unsuccessful NFB training (Wiech and Tracey, 2009).

Correlation analysis across all participants (*n* = 45) further showed that a greater training efficacy predicts a greater increase in pain thresholds, particularly at hand contralateral to NFB target site. Theoretically, this finding provides causal evidence between sensorimotor cortical excitability and sensory-discriminative aspect of pain. Also, it highlight that the variability in the NFB training efficacy should be noted with cautious. Previous studies reported that the degree of change in target brain function and behavior depends on NFB training efficacy (Kober et al., 2018, Kober et al., 2015b, Ros et al., 2010, Ros et al., 2013). NFB training induced psychophysiological benefits could only be observed among learners who could successfully self-regulate their brain activity (Hanslmayr et al., 2005, Hsueh et al., 2016). In terms of pain modulation, attenuation in clinical pain after NFB training was found among patients who could successfully self-regulate their sensorimotor α-oscillations (Al-Taleb et al., 2019, Vuckovic et al., 2019). Therefore, personalized and optimized training protocols that minimize individual variability would hopefully increase pain modulation effect. As previous studies have well documented a close association between sensorimotor γ-oscillations and pain perception (Gross et al., 2007, Hu and Iannetti, 2019, Zhang et al., 2012), future studies can also consider the application of NFB training protocols targeted on regulating sensorimotor γ-oscillation for pain modulation, or simultaneously regulating sensorimotor α-oscillation and γ-oscillation.

### 4.3 Limitations

Despite the implications, there were several limitations that should be acknowledged in the present study. First, we only recorded EEG signals at sensorimotor electrodes (C3 and C4) during the NFB training session due to the equipment limitation. Future studies should use whole-brain EEG equipment to assess whether pain modulation can be predicted by NFB training efficacy either specifically at the target site or more generally at global sites (e.g., frontal or parietal electrodes). Second, we only quantified pain perception by comparing psychophysical measurements of pain, including pain thresholds and pain ratings to identical painful stimuli before and after NFB training. How the NFB training efficacy influences neuronal responses to painful stimuli remains unclear. Third, since all participants received real NFB training protocols, we cannot definitively conclude that the change of pain measurements after NFB training (e.g., increased pain thresholds and increased pain unpleasantness ratings) was arisen from the specific neurophysiological processes of NFB training or other non-specific factors (such as psychosocial factors). Future studies can adopt a double-blind sham-controlled protocol to solve this problem. Nevertheless, NFB training efficacy in the present study was individually quantified using the regression slope of α-oscillation LI that indicates the α-oscillation amplitude change throughout the training blocks specifically at target site relative to the nontarget site. Pain measurements before and after NFB training session were compared between high- and low-efficacy group, which allowed assessing whether pain modulation was dependent upon NFB training efficacy. The increased pain thresholds after NFB training was selectively applicable to high-efficacy group, and NFB training efficacy can predict the amount of increase in pain threshold, particularly at hand contralateral to NFB target site. The inclusion of nontarget site as a control site, the between-group comparisons, as well as across-participant correlations, could strengthen support for the conclusion that the increase in pain thresholds after NFB training largely reflected a real NFB training effect. In contrast, whereas the increase in pain ratings after NFB training was selectively observed in the low-efficacy group, NFB training efficacy could not predict the amount of increase in pain ratings. The increase in pain ratings after NFB training thus could be resulting from other non-specific, psychosocial factors such as negative mood and emotion.

## 5 Conclusion

To sum up, we have shown that NFB training overall decreased the sensory-discriminative aspect of pain, but increased the affective-motivational aspect of pain, both of which modulation was dependent upon NFB training efficacy. In addition, a greater NFB training efficacy predicts a greater increase in pain thresholds, thus providing more causal evidence for the link between sensorimotor α-oscillations and the sensory-discriminative aspect of pain perception. The finding highlights the influence of inter-individual variability in NFB training efficacy on subsequent pain modulation. Future studies can optimize training protocols for individuals that cannot successfully self-regulate their α-oscillations.

## Funding

This study was supported by the 10.13039/501100001809National Natural Science Foundation of China (31871127 and 81901830), the Humanity and Social Science Youth Foundation of the Ministry of Education in China (19YJC190018), the Features Innovative Projects of Guangdong Province Ordinary University (2019KTSCX149), the 10.13039/501100017607Shenzhen Basic Research Project (JCYJ20190808154413592), the postgraduate innovation development fund project of 10.13039/501100009019Shenzhen University (315-0000470535), the 10.13039/100015549Science and Technology Development Fund, Macau SAR (Grant no. 055/2015/A2 and 0045/2019/AFJ) and the University of Macau Research Committee (MYRG projects 2016-00240-FST and 2017-00207-FST). The Wellcome Centre for Integrative Neuroimaging is supported by core funding from the 10.13039/100010269Wellcome Trust (203139/Z/16/Z).

## 7 Data availability statement

Data and code supporting the findings of this study is available from the corresponding author upon reasonable request.

## CRediT authorship contribution statement

**Weiwei Peng:** Conceptualization, Methodology, Formal analysis, Writing - original draft, Funding acquisition. **Yilin Zhan:** Investigation, Formal analysis. **Yali Jiang:** Investigation, Formal analysis. **Wenya Nan:** Conceptualization, Methodology, Formal analysis, Writing - original draft, Funding acquisition, Supervision. **Roi Cohen Kadosh:** Conceptualization, Writing - review & editing. **Feng Wan:** Conceptualization, Software, Resources.

## Declaration of Competing Interest

The authors declare that they have no known competing financial interests or personal relationships that could have appeared to influence the work reported in this paper.
