# Supplementary material for: Individual variation in alpha neurofeedback training efficacy predicts pain modulation
Source: Neuroimage Clin. 2020 Sep 29;28:102454. doi: 10.1016/j.nicl.2020.102454 (PMC7566954; doi:10.1016/j.nicl.2020.102454)
Supplement: Supplementary data 1 [file mmc1.docx]

**Supplementary Materials for “Individual variation in alpha neurofeedback training efficacy predicts pain modulation”**

**
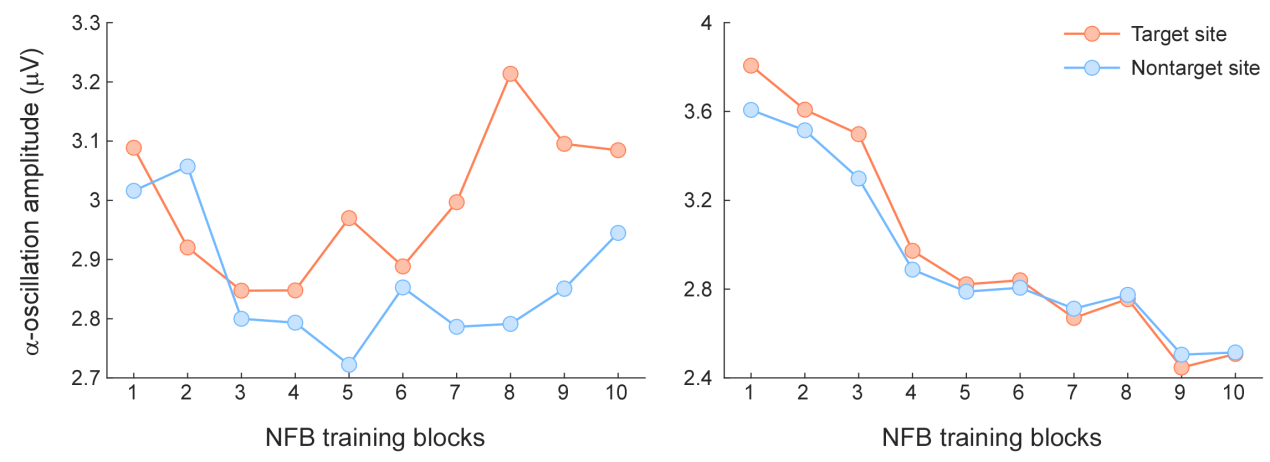
**

**Figure S1.** The sensorimotor α-oscillation amplitudes throughout the NFB training blocks (1-10) at target and nontarget sites for two participants with relatively high (left) and low (right) NFB training efficacy

**Table S1.** Statistical comparisons of pain measurements from all participants (*n* = 45)

|  | Pain threshold (J) | | Pain intensity | | Pain unpleasantness | |
| --- | --- | --- | --- | --- | --- | --- |
|  | *F_1,44_* | η_p_^2^ | *F_1,44_* | η_p_^2^ | *F_1,44_* | η_p_^2^ |
| Stimulation Site | 0.01 | <0.001 | 0.27 | 0.01 | 0.33 | 0.01 |
| Session | 25.51*** | 0.38 | 2.04 | 0.04 | 7.24** | 0.14 |
| Stimulation site × Session | 1.98 | 0.04 | 0.75 | 0.02 | 0.04 | 0.001 |

Notes: A two-way repeated measures ANOVA with factors of Stimulation Site (contralateral or ipsilateral to the target site) and Session (pre- or post-NFB session) was applied to pain measurements. **: *p* < 0.01; ***: *p* < 0.001.

**Table S2.** Statistical comparisons of pain measurements from two groups of participants with high and low NFB training efficacy (*n* = 12 for each group)

|  | Pain threshold (J) | | Pain intensity | | Pain unpleasantness | |
| --- | --- | --- | --- | --- | --- | --- |
|  | *F_1,22_* | η_p_^2^ | *F_1,22_* | η_p_^2^ | *F_1,22_* | η_p_^2^ |
| Group | 2.23 | 0.09 | 0.74 | 0.03 | 2.69 | 0.11 |
| Stimulation Site | 0.13 | 0.01 | 0.27 | 0.01 | 0.13 | 0.01 |
| Session | 9.95** | 0.31 | 0.32 | 0.01 | 5.56* | 0.20 |
| Group × Stimulation Site | 0.34 | 0.02 | 0.01 | <0.001 | <0.01 | <0.001 |
| Group × Session | 5.45* | 0.20 | 8.01** | 0.27 | 3.42 | 0.14 |
| Stimulation Site × Session | 0.93 | 0.04 | 0.05 | <0.001 | 0.31 | 0.01 |
| Group × Stimulation Site × Session | 0.19 | 0.01 | 0.30 | 0.01 | 0.04 | <0.001 |

Notes: A three-way repeated measures ANOVA with a between-participant factor of Group (high- vs. low-efficacy group), and two within-participant factors of Stimulation Site (contralateral vs. ipsilateral to target site) and Session (pre- vs. post-NFB session) was applied to pain measurements. *: *p* < 0.05; **: *p* < 0.01.
